# Risk of Hormone Escape in a Human Prostate Cancer Model Depends on Therapy Modalities and Can Be Reduced by Tyrosine Kinase Inhibitors

**DOI:** 10.1371/journal.pone.0042252

**Published:** 2012-08-06

**Authors:** Charlotte Guyader, Jocelyn Céraline, Eléonore Gravier, Aurélie Morin, Sandrine Michel, Eva Erdmann, Gonzague de Pinieux, Florence Cabon, Jean-Pierre Bergerat, Marie-France Poupon, Stéphane Oudard

**Affiliations:** 1 Translational Research Department, Institut Curie, Paris, France; 2 Signaling and Prostate Cancer Group, Université de Strasbourg, Strasbourg, France; 3 Biostatistics Department, Institut Curie, Paris, France; 4 U900, INSERM, Paris, France; 5 Ecole des Mines de Paris, ParisTech, Fontainebleau, France; 6 FRE2944, CNRS, Villejuif, France; 7 Biomarker Research and Validation Department, BioMérieux, Marcy l’Etoile, France; 8 Anatomie et Cytologie Pathologiques, Hôpital Trousseau, Tours, France; 9 Medical Oncology, Hôpital Européen Georges Pompidou, Paris, France; 10 Université Paris V René Descartes, Paris, France; Florida International University, United States of America

## Abstract

Almost all prostate cancers respond to androgen deprivation treatment but many recur. We postulated that risk of hormone escape -frequency and delay- are influenced by hormone therapy modalities. More, hormone therapies induce crucial biological changes involving androgen receptors; some might be targets for escape prevention. We investigated the relationship between the androgen deprivation treatment and the risk of recurrence using nude mice bearing the high grade, hormone-dependent human prostate cancer xenograft PAC120. Tumor-bearing mice were treated by Luteinizing-Hormone Releasing Hormone (LHRH) antagonist alone, continuous or intermittent regimen, or combined with androgen receptor (AR) antagonists (bicalutamide or flutamide). Tumor growth was monitored. Biological changes were studied as for genomic alterations, AR mutations and protein expression in a large series of recurrent tumors according to hormone therapy modalities. Therapies targeting Her-2 or AKT were tested in combination with castration. All statistical tests were two-sided. Tumor growth was inhibited by continuous administration of the LH-RH antagonist degarelix (castration), but 40% of tumors recurred. Intermittent castration or complete blockade induced by degarelix and antiandrogens combination, inhibited tumor growth but increased the risk of recurrence (RR) as compared to continuous castration (RR_intermittent_: 14.5, RR_complete blockade_: 6.5 and 1.35). All recurrent tumors displayed *new* quantitative genetic alterations and AR mutations, whatever the treatment modalities. AR amplification was found after complete blockade. Increased expression of Her-2/neu with frequent ERK/AKT activation was detected in all variants. Combination of castration with a Her-2/neu inhibitor decreased recurrence risk (0.17) and combination with an mTOR inhibitor prevented it. Anti-hormone treatments influence risk of recurrence although tumor growth inhibition was initially similar. Recurrent tumors displayed genetic instability, AR mutations, and alterations of phosphorylation pathways. We postulated that Her-2/AKT pathways allowed salvage of tumor cells under castration and we demonstrated that their inhibition prevented tumor recurrence in our model.

## Introduction

Androgen receptor (AR) controls cell proliferation and survival in the normal prostate and prostate carcinomas (PCa). Thus androgen deprivation is first-line treatment of PCa. Hormone therapy includes castration pharmacologically achieved with luteinizing-hormone releasing hormone (LH-RH) agonists or antagonists, AR antagonists as flutamide or bicalutamide or new treatment modalities such as inhibitor of 17–20 lyase (abiraterone acetate, TAK700) or MDV3100 [1]. Treatments are given continuously or intermittently, by LH-RH inhibitor monotherapy, antiandrogen monotherapy or combined as referred to complete androgen blockade.

Whatever the hormone therapy, most tumors respond then acquire androgen independence and recur [2], [3]. Several mechanisms have been proposed [4], [5]. *i.* Genomic changes occur during tumor progression but their role remains unclear, although clonal chromosome abnormalities have been found in PCa [6], [7]. *ii.* Alteration of AR expression is frequent due to gene amplification [8], increased transcription, or stabilization of the AR protein via phosphorylation of specific AR residues [9], [10], AR mutations that broaden the ligand spectrum [8], alterations in nuclear receptor coactivators, and ligand-independent binding of AR to DNA [2], [11]. The prevalence and influence of AR alterations on disease progression are not known because of the variability in treatment regimens, limited access to material from patients and thus few comprehensive sequencing studies. *iii.* Activation of survival pathways is involved in hormone escape [12], such as Her-2/neu (a growth factor receptor tyrosine kinase), mTOR/AKT (target of rapamycin/AKT), or ERK1,2 (extracellular-signal-regulated kinase), all implicated in AR phosphorylation [5], [10]. Her-2/neu expression is usually low in PCa. However, high levels of Her-2/neu were found associated with shortened survival times in a subset of PCa patients [13], [14]. More, Craft *et al.* showed that forced Her-2/neu expression modulates AR signaling and leads to androgen independence [15]. An altered AKT pathway was associated with PCa progression and the emergence of AI tumors [16]. Moreover, Graff *et al.* showed that forced overexpression of AKT in LNCaP cell line accelerated tumor growth [17]. AKT might be an alternative way by which Her-2/neu leads to outlaw AR activation [18].

A key question in clinics is whether modalities of hormone treatment differently affect the risk of escape. To respond to this critical question, we used an experimental model of a hormone dependent of human prostate cancer (PAC120), derived directly from a patient and growing in immunodeficient mice. We evaluated the effect of different hormone treatment modalities on the immediate response and on the risk of recurrence; the biological changes associated with different treatments, as genome alterations, *AR* mutations, and growth factor expression/activation were studied. The involvement of phosphorylation pathways in hormone escape led us to test combination of tyrosine kinase inhibitors with pharmacological castration to reduce the risk of tumor recurrence.

## Methods

### Prostate Tumor Xenografts

PAC120, a hormone-dependent human-in-mouse PCa xenograft, [19] maintained by serial transplantation into the interscapular fat pad of male Swiss nude mice (Crl:NU(Ico)-Foxn^lnu^) from Charles River (L’Arbresle, France) was used between passages 47 and 51. Tumor pieces of 20 mm^3^±5 (±20. 10^6^ cells) where transplanted. All protocols followed institutional guidelines as put forth by the French Ethical Committee.

### Treatments

degarelix (Firmagon® known as FE 200486 during it development, Ferring Research Institute Inc., San Diego, CA) [20] injected subcutaneously monthly at 10 mg/kg [19], bicalutamide (Casodex®, Astra Zeneca, France) and flutamide (Eulexine®, Schering-Plough, Kenilworth, N.J.) given at 50 mg/kg, per os, 5 days per week. Trastuzumab (Herceptin®, Roche, France) injected weekly at 10 mg/kg via intraperitoneal administration. Everolimus (Afinitor®, Novartis Pharma AG, Switzerland) given per os at 2 mg/kg, 3 days per week. We define continuous castration as injection of degarelix alone once a month. Mice were kept under treatment until the relapse of the tumor (tumor had to reach a 1500 mm^3^). Intermittent castration consisted of 2 injections of degarelix at day 0 and 28 (one cycle), then stopped the treatment until the tumor reach back 2 times the initial volume. The complete androgen blockade is the association of degarelix monthly in combination with the anti-androgen receptor inhibitor (either bicalutamide or flutamide). Figure S1 represents a schematic diagram of the treatments modalities.

### Tumor Growth Assessment

Follow-up of tumor growth was done according to Marangoni et al. [21]. Recurrence was defined as the time in days required for tumors to achieve a 5-fold increase from initial volume.

PAC120 xenograft tumor fragments were randomly implanted into 204 male mice. As soon as tumors reached a mean volume of 63–250 mm^3^, mice were assigned to treatment (*n* indicated in Table S1). After tumor regrowth, AI tumors were transplanted into pharmacologically castrated male mice and characterized at early passages (1 to 4).

### RT-PCR mRNA Analysis

Total RNA was extracted as previously published [21]. Sequences of primers are indicated in the Methods S1.

### Functional AR Mutation Detection by ADE2 Reporter Assay

We used a functional assay in the EJ250 yeast strain to detect AR mutations [22].

#### Androgen receptor (AR) yeast-based activity assay

An AR activity assay performed in the recombinant EJ250 yeast strain (MATa ade2-101 his3-D200 leu2-D1 lys2-801 trp1-D1 ura3-52 URA3: ARE-ADE2[pRS/AREAde2]) was used to screen tumor samples for AR mutations as previously described [22], [23]. In this EJ250 yeast strain, the expression of the ADE2 reporter gene, necessary for adenine biosynthesis, was placed under the tight control of an androgen-dependent promoter [22]. Consequently, EJ250 yeast growth relies on the transcriptional activity of a ligand-activated androgen receptor. After transformation with an AR expression plasmid as described earlier [22], yeast were plated on selective media depleted of adenine and containing either dihydrotestosterone (DHT), dehydroepiandrostenedione, β-estradiol, progesterone, 17á-hydroxyprogesterone, flutamide or cyproterone acetate (Sigma Aldrich, St Quentin Fallavier, France) at the indicated concentration. A negative control (vehicle) and a positive control (supplemented with adenine) were also included. When applied to tumor samples, mutated AR variants can be distinguished from wild type AR according to their response to the steroid or nonsteroid molecule added in the medium.

#### Detection of androgen receptor (AR) mutations in tumor samples

Briefly, total RNA was prepared from tumor samples (RNeasy Midi Kit, Qiagen, Courtaboeuf, France). Reverse transcription was performed with the Omniscript reverse transcription kit (Qiagen) from 1 µg of total RNA. AR cDNA fragments were amplified with the forward (5′-TGCGGCGGCGCAGTGCCGCTAT-3′) (nucleotides 2310–2339) and reverse (5′-GGTGCCATGGGAGGGTTAGATAGGGAG-3′) (nucleotides 4075–4101) primers flanking exons 1–8 (GenBank NM_000044.2, nucleotides 2310–4101). Thereafter, 100 ng AR cDNAs fragments were inserted into a yeast expression “gap repair” plasmid by homologous recombination, and after plating and incubation at 30°C for 72 h, yeast colonies were scored [22]. The wild-type AR was assayed in parallel as experimental control. The androgen receptor-dependent yeast growth in response to the steroid or antiandrogen added to the medium was evaluated by colonies scoring. Data are presented as histograms corresponding to the ratio of the number of yeast colonies scored to the number of colonies obtained in the presence of 100 nM DHT. For AR mutations characterization, the AR expression plasmid was rescued from yeast cells for sequencing. Briefly, yeasts were lysed with 150 to 212 µm acid-washed glass beads (Sigma Aldrich) and plasmid DNA was extracted with the Nucleospin plasmid kit (Macherey-Nagel, Hoerdt, France) and processed for sequencing (GATC Biotech AG, Konstanz, Germany).

### Protein Expression Analyzed by Western Blotting

AKT, phospho-AKT (S473), ERK1/2 (Cell Signaling Technology, Ozyme, Saint Quentin en Yveline, France), and PSA (Dako) were used at 1∶1000. Her-2/neu (Cell Signaling), AR (Upstate, Millipore, Saint Quentin en Yveline), and p-ERK1/2 (RnD systems, Lille, France) were used at 1∶500. Anti-beta-actin was used at 1∶5000 (Sigma-Aldrich, Saint Quentin Fallavier, France). Protocols are detailed in Methods S1.

### Comparative Genomic Hybridization Arrays

Procedure was previously published [24]. We performed co-hybridization between the DNA extracted from AI variants and those from PAC120p47. Minimal common alterations were identified [25]. Hierarchical clustering was performed on profiles based on minimal regions. Euclidian distance was used as the similarity measure and the Ward algorithm as the agglomerative method. Separation into groups was proposed on the basis of the structure of the dendrogram.

### Histology

Light microscope histology studies used 4-µm-thick formalin fixed paraffin-embedded sections from PAC120, PAC120 residual tumors and AI variants stained with hematoxylin and eosin safran.

### Statistical Analysis

We used the Mann-Whitney test, Kaplan-Meier survival plots, log-rank tests, and the Fisher’s exact test, according to respective experiments as detailed in Methods S1.

## Results

### *In vivo* Response to Hormone Treatment Regimens

**Figure 1:**
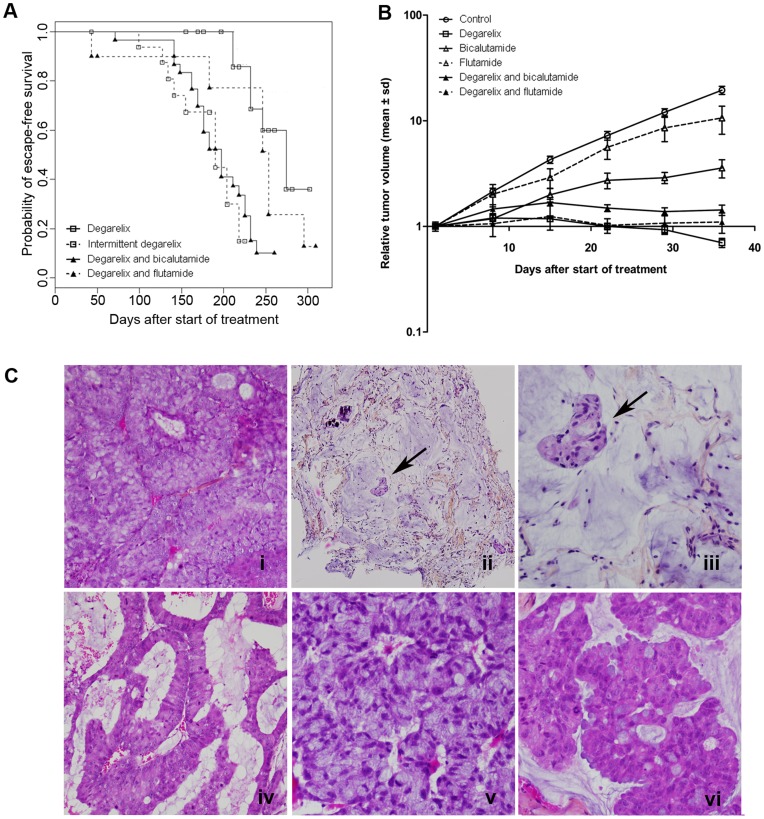
PAC120 xenograft tumor response to androgen deprivation treatment regimens. **A**) Kaplan-Meier analysis of hormone escape-free survival after continuous (white square, full line), intermittent (white square, dotted line) degarelix, bicalutamide plus degarelix (black triangle, full line) or flutamide plus degarelix (black triangle, dotted line). **B**) Tumor growth curves as a function of time in control (white cercle, full line), bicalutamide (white triangle, full line), flutamide (white triangle, dotted line),degarelix (white square, full line), bicalutamide plus degarelix (black triangle, full line) and flutamide plus degarelix (black triangle, dotted line); **C** i) PAC120 xenograft [x200], residual PAC120 tumor (300 days) ii)[x100], iii) [x400], AI variants with adenoid features (iv) [x100], amphicrine features (v) [x200], and mucosecretant features (vi) [x200].

Continuous castration by degarelix arrested the growth of all PAC120 tumors. Recurrences were observed in 8/20 mice, in a median delay of 274 days [range 211–323] (Figure 1A). Intermittent castration was tested according to the following protocol: The first administration of degarelix reduced tumor volume in all animals (14/14, 100%). The second administration was given once tumor regrew of two-fold in volume, in a median delay of 119 days. Six tumors such treated did not respond (6/14, 43%) and 8 showed low response or stabilization. Kaplan Meier analysis showed an increased relative risk of recurrence of 14.5 (95% CI: 2.98–70.9, *p*<0.001, log-rank test) (Figure 1A and Table S2). Therefore, intermittent androgen deprivation increased the risk of recurrence and reduced time to progression as compared with continuous castration.

Flutamide or bicalutamide significantly delayed tumor growth (*p = *0.03, and *p*<0.001, respectively) (Figure 1B). Complete androgen blockade (degarelix plus bicalutamide) arrested tumor growth in 30 treated cases but 24 tumors recurred. Recurrences occurred earlier than after castration alone (Table S2) with increased relative risk of recurrence (relative risk of 6.55; 95% CI: 2.41–17.8, *p*<0.001, log-rank test) (Figure 1A and Table S2). Complete androgen blockade with degarelix plus flutamide led to 8/11 recurrent tumors and a median time to hormone escape of 253 days [range 43–337] (Table S2); increase of recurrence risk was not statistically significant as compared with degarelix alone (*p = *0.561, log-rank test).

### Histological and Molecular Alterations Associated with Androgen Independence

Residual PAC120 tumors under castration showed necrotic areas, numerous mitoses and no apoptotic bodies (Figure 1C *ii & iii*). Androgen independent (AI) variants displayed histological changes, such as mucinous, neuroendocrine-like, or adenoid differentiation features (Figure 1C iv–vi). All AI variants displayed mixed patterns, dominated by mucinous differentiation or/and apocrine differentiation and numerous multinuclear cells. None features were specific to any particular hormone treatment.

**Figure 2:**
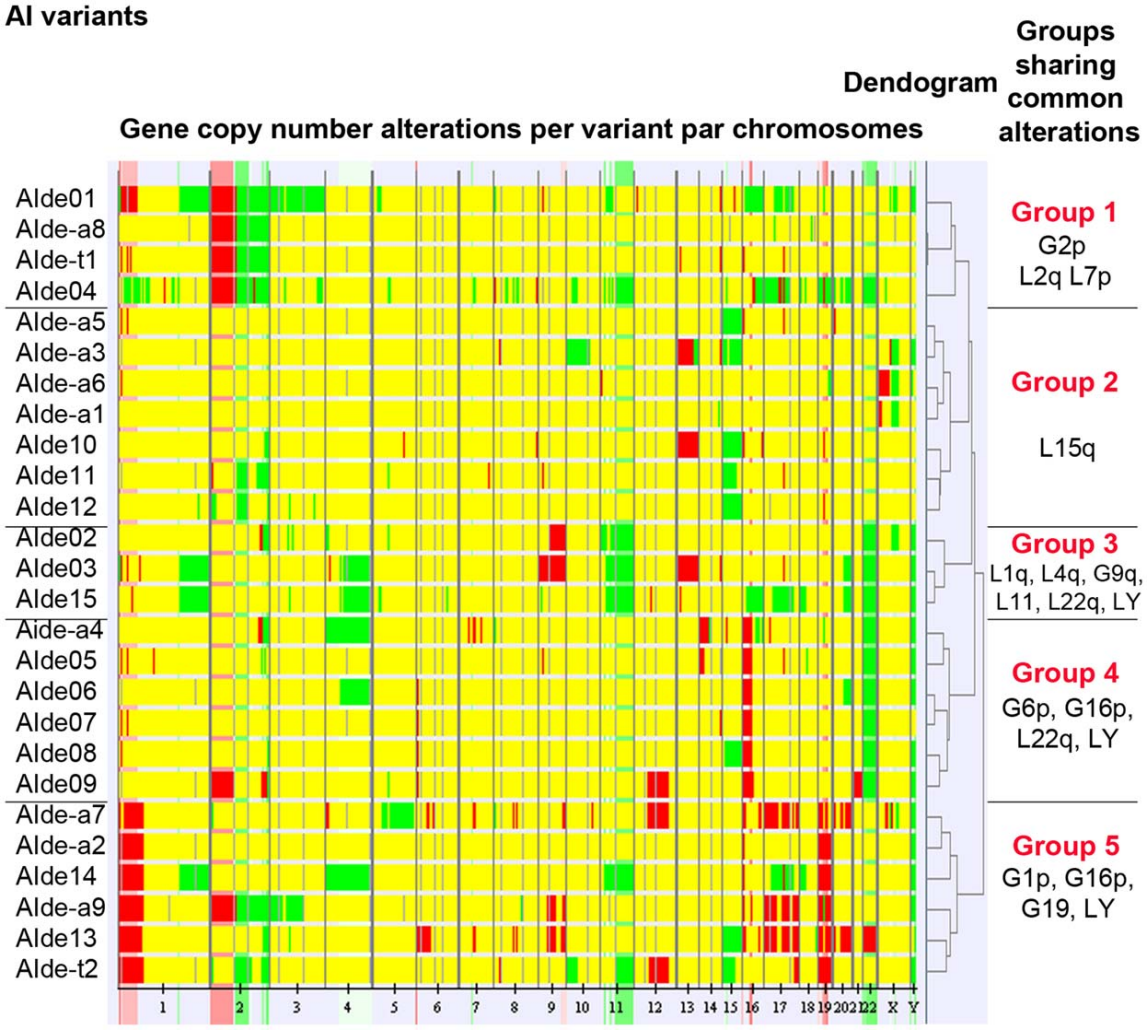
Unsupervised hierarchical clustering analysis on gene copy number alterations. There was no relationship between gene copy number alterations and the type of hormone treatment. The left hand column indicates numbered AI variants by treatment where *de* denotes degarelix, *de-a* degarelix plus antiandrogen (bicalutamide or flutamide), *de-t* degarelix plus trastuzumab. Green and red colors correspond to losses and gains of gene copy number, respectively. Yellow corresponds absence of difference in terms of gene copy number between AI variants and parental PAC120 DNA.

The chromosomal stability of PAC120 tumors through time and passages has been previously demonstrated [24]. We co-hybridized AI DNA with the parental PAC120 DNA to reveal the new alterations. Thirty-five minimal regions were altered in at least 5 (20%) of the 26 AI variants studied. Surprisingly, none of the alterations were common to all AI variants. Gene copy gains and losses are detailed in Table S3. The 26 AI variants were grouped by altered regions into five distinct groups using a hierarchical cluster analysis (Figure 2). However, we did not find any treatment-specific pattern of alterations associated with recurrences (Fisher exact test and Benjamini and Hochberg correction to adjust for multiple testing). These results show that androgen deprivation generated a random diversity of new genetic alterations but no specific pattern could be attributed to the type of treatment regimen. These alterations were stably acquired (data not shown).

*AR* gene amplification was observed in 15% (4/26) of AI variants and occurred only in the AI derived after complete blockade (AIde-a) group (Table S3). Frequency of gene amplification after complete androgen blockade was significantly higher than after castration alone (*p = *0.01, Fisher exact test). In these cases AR mRNA levels were increased of 10 to 20-fold, as were intratumoral PSA mRNA levels (Figure S2).

**Figure 3:**
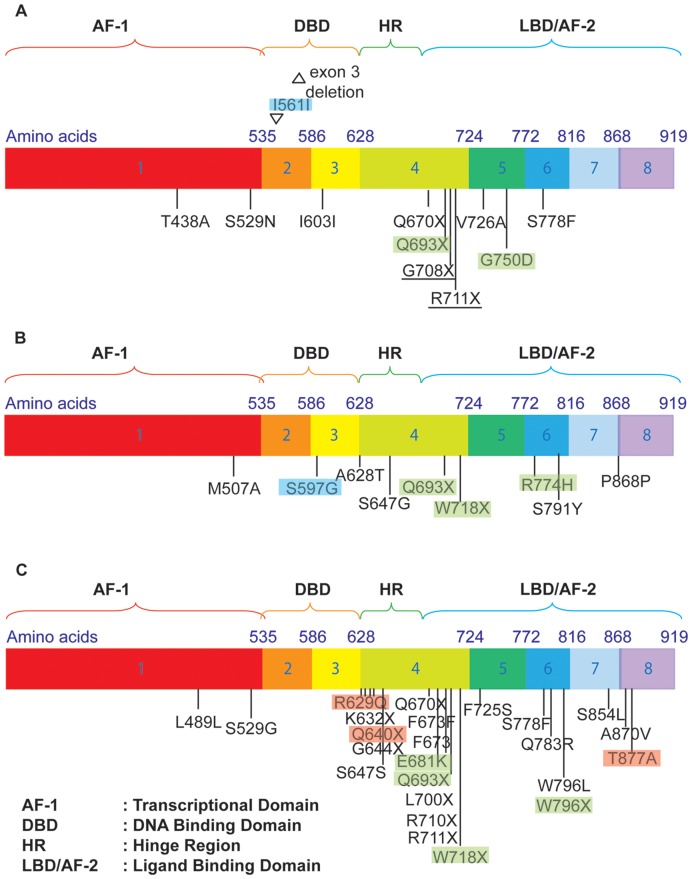
AR gene sequence showing mutations detected in AI variants A) after treatment with single-agent degarelix, B) after antiandrogens and C) after complete androgen blockade. Full underlines show mutations found in more than one tumor treated with the same agent. Mutations depicted with a blue, green or red background have previously been described in PAIS, CAIS and PCa, respectively.

*AR* mutations were detected using a functional yeast-based assay as described previously [22]. No functional *AR* mutations were found in the PAC120 tumor but appeared in 28% (10/36) of AI variant tumors. An example of the profile that we obtained for AIde-a4 is shown in the Figure S3. Mutations occurred in 46%, 17%, and 25% after complete blockade, antiandrogens and degarelix, respectively (Table S4). AR cDNA sequencing revealed 30 different *AR* mutations (Figure 3, Table 1), of which 70% were located in the hinge region (HR) or in the ligand binding domain (LBD), 27% led to a truncated AR protein. Some AI variants displayed several mutations. Only Q693X was common to all of the different hormone treatments. The Q693X mutation, as well as the other premature stop codons in Table 1, leads to a truncated AR variant lacking the ligand-binding domain and AF-2. The Q693X AR variant has also been detected in human prostate cancer tumors (unpublished data). Truncated AR variants demonstrate constitutive transcriptional activities as previously reported [23]. They have been associated with castration-resistance as they can promote prostate cancer cell growth in vitro cell cultures and animal tumor models in the absence of androgens (Figure S4). Most mutations were novel.

**Table 1:** Classification of androgen receptor mutations detected.

Functional consequences	Mutations
**Constitutively active AR variants**	K632X; Q640X; G644X; Q670X; Q693X; L700X; G708X; R710X; R711X; W718X; W796X
**Promiscuous AR variants**	T877A
**Unknown functions**	T438A; M507A; S529G; S529N; R629Q; S647G/S791Y; F673I; F725S; V726A; Q738R; G750D; R774H; S778F; W796L; S854L; A870V

**Figure 4:**
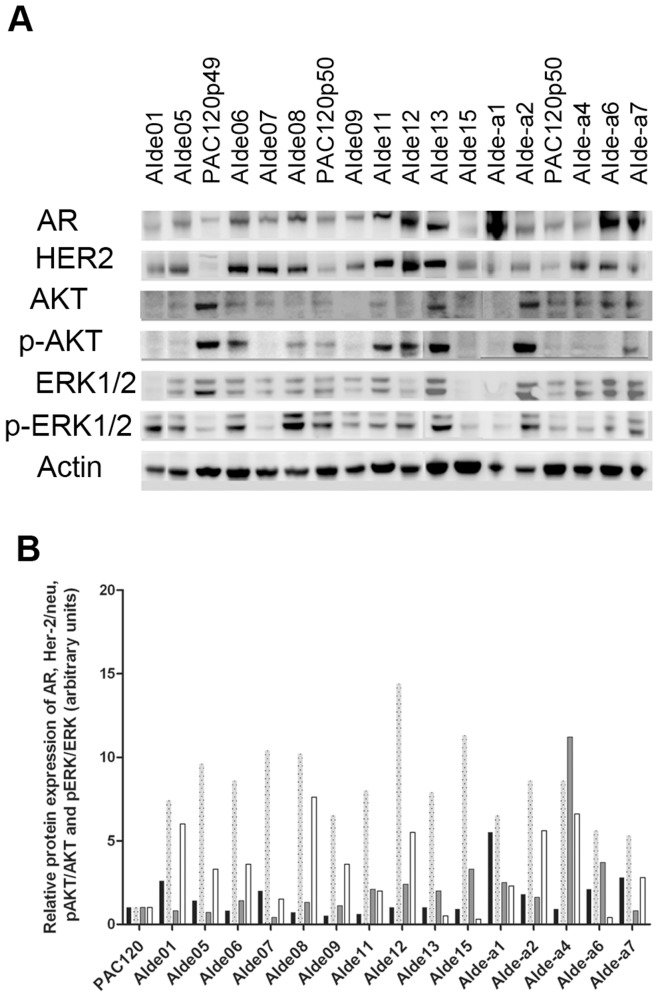
Protein expression of the androgen receptor (AR), human epidermal growth factor receptor 2 (Her-2/neu) and ratios of pAKT/AKT and pERK/ERK detected using A) Western blotting analysis in androgen-independent (AI) variants. Actin was used as a control for loading protein. **B**) Histograms representing the quantification of AR, Her-2/neu and ratios of pAKT/AKT and pERK/ERK normalized to actin and mean of PAC120 coming from various passages (expressed arbitrary units). AR (black bars) was expressed in PAC120 and all AI variants and Her-2/neu (dotted grey bars) was overexpressed in all AI variants. Either p-AKT/AKT (grey bars with full line) or pERK/ERK (white bars) were activated in AI variants. Abbreviations used to identify variants are explained in the legend of Figure 2.

The AR protein level (normalized by actin) was increased in all AI variants (Figure 4). These data suggest that AR alterations are key events in tumor recurrence after androgen deprivation, required for prostate cancer cell survival.

Her-2/neu protein was overexpressed in all AI variants (*n = *17) tested as compared with PAC120 (Figure 4). In addition, AKT or ERK1,2 or both pathways were activated in the majority of AI variants, showing that such an activation is associated with escape to hormone deprivation, and participates compensatory mechanisms of AR activation. The key role of these phosphorylation pathways was indirectly demonstrated by the strong effect of their inhibition.

### Effects of Combination Castration with Tyrosine Kinase Inhibitors

**Figure 5:**
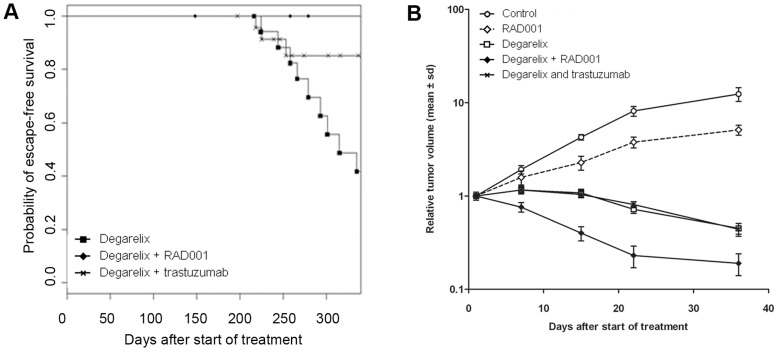
PAC120 xenograft tumor response to combination treatment of castration plus tyrosine kinase inhibitors shown as A) escape-free survival (Kaplan-Meier analysis) where the median time to hormone escape after continuous treatment with degarelix (black square, full line) was 274 days and after combination of trastuzumab plus FE 200846 (cross, full line) was 351 days. **B**) tumor growth curves as a function of time in the control group (white cercle, full line) and in groups receiving everolimus 2 mg/kg three times a week (white diamond shape, dotted line), degarelix 10 mg/kg monthly (white square, full line), trastuzumab plus degarelix (cross, full line) or everolimus plus degarelix (black diamond-shaped, full line).

Degarelix combined with trastuzumab significantly and drastically reduced the relative risk of recurrence to 0.17 (95% CI: 0.04–0.67, *p = *0.005, log-rank test) (Figure 5A), and prolonged the median time to hormone escape from 274 to 351 days (Table S2) versus castration with degarelix alone. Tumor recurrence was observed in only 17% (4/24) of animals receiving the combination treatment. Trastuzumab as a single agent had no effect on the tumor growth.

Degarelix combined with everolimus completely prevented hormone escape of the 17 tumors treated. As single-agent everolimus delayed tumor growth and the TGI_36 days_ was 59% (*p = *0.001, Mann-Whitney) (Figure 5B).

These results demonstrate a superior effect of castration when combined with HER-2/neu targeting antibodies or mTOR inhibitor.

## Discussion

We conducted an *in vivo* experimental study of the effect of different hormone treatments on the risk of hormone escape. We established a large series of AI variants to study the diversity of genetic and molecular events occurring after hormone escape. We used an hormone-dependent human PCa xenograft (PAC120) [19]. PAC120 derived directly from a primary tumor of a patient. PAC120 showed striking karyotype stability, maintained stable hormone dependence [24] and lack of *AR* mutations, after multiple passages [19].

Continuous pharmacological castration arrests tumor growth, escapes occur in 40% of cases. Antiandrogen monotherapy slowed down tumor growth. This reflects the clinical observations. In our model, complete androgen blockade increased the risk of hormone escape, as compared with castration alone. It seems that complete androgen blockade exerts a high pressure on the AR signaling pathway, leading to the activation of several survival pathways. Large randomized studies and meta-analyses showed that complete blockade did not prolong the time to progression in most patients [26]. Phase II and III studies comparing intermittent versus continuous castration in prostate cancer were associated with a higher risk of hormone escape in case of intermittent castration, but overall survival was not shortened after intermittent androgen deprivation in comparison with a continuous schedule with a better quality of live and less cost [27], [28]. The discrepancy with our data could be explained by the aggressiveness of PAC120 (Gleason 9), whereas the clinical studies included a large diversity of prostate tumors of low and high grade. More, the time chosen to restart the treatment was based on a unic parameter, i.e. tumor volume, while in clinic several parameters are considered (testosteronemia, PSA). Intermittent therapy might be not appropriate to all cases of prostate cancers.

AI variants displayed biological changes. Histology of tumors under castration showed numerous mitosis and necrotic areas, suggesting a balance between mitosis/cell survival/death and dismissing any dormant state of tumoral residue. AI variants displayed mixed patterns, mucinous or/and apocrine differentiation similar to the parental tumor and none features specific to hormone treatment modality.

We checked that the deeply altered karyotype of PAC120 remained quite stable through transplantation and time [24], and we showed that castration resulted in a wave of new genomic alterations. More, these acquired alterations are stable (data not shown) perhaps explaining why hormone independence was irreversibly acquired. Surprisingly none of the new alterations were common to the 26 AI variants. Genetic instability is known to be linked to cancer progression, our observation suggests such a progression mechanism [29], [30]. Then we searched for alterations involving AR expression, gene amplification or genetic mutations. *AR* gene amplifications, were detected after complete androgen blockade as found in patients [31]. *AR* mutations which contribute to hormone escape by providing a larger receptivity of AR to other ligands [8], affect several AR-dependent processes [2], [32]. Steinkamp *et al.* reported that hormone-naïve PCa tumor samples had very few *AR* mutations suggesting that these mutations offered little growth advantage and were in fact random “passenger” mutations [33]. The assay we used specifically selects for *AR* mutations that affect receptor function [22]. We observed a huge diversity of androgen deprivation therapy-induced *AR* mutations, although very few were common to all AI variants and a majority were not described [34]. Altogether, AI variants illustrated the putative diversity of biological events associated with hormone escape and underlined that an AR-dependent control remains central in prostate cancers. Novel therapeutic approaches targeting the AR pathways by inhibiting either the 17–20 lyase or the translocation of the receptor towards the nucleus underlined this key point [1]. Our last set of data is another alternative of blocking AR pathways that could be addressed in clinics.

We examined Her-2/neu and p-AKT expression in PAC120 AI variants to determine whether kinase and phosphorylation pathways were involved in the hormone escape, as shown by others [15], [18]. In clinical samples, Her-2/neu expression was elevated in AI tumors [14], [35] and reported as an early event in the androgen dependence to independence switch [36]. The proposed mechanism for the role of Her-2/neu in hormone escape is that this kinase activates AR phosphorylation (via the MAPK pathway or AKT pathway) [18], [37], which in turn maintains AR integrity and thus, its function in absence of testosterone. Lin HK et al., showed that PI(3)K/Akt, but not PI(3)K/p70S6K signaling pathway can modulate the AR transactivation. By using herceptin, we block the PI(3)K pathway upstream which could explain reduction of tumor escape [38].

Other alterations can contribute to a ligand-independent activation of AR. Loss of PTEN, PI3K mutation, AKT1 and AKT2 amplification may activate AKT kinase. Increase of phospho-AKT levels was detected in bicatulamide-treated PCa [39]. In our AI variants, Her-2/neu and p-AKT expression/activation was increased and the preventive effect of trastuzumab- or everolimus- degarelix combined treatments on hormone escape demonstrates their key role. The Her-2/AKT and mTOR pathways are targets accessible to new inhibitors; trastuzumab has demonstrated its efficacy in Her-2/neu-amplified breast cancer [40]. Wang et al demonstrated on prostate cancer cell line that inhibition of mTORC1 and AR pathways by a combination of rapamycin and bicatulamide led to apoptosis and could be a therapeutic value in prostate cancer treatment [41]. A recent study showed on LnCaP and a short term experiment the benefit of the blockade of AR signaling in combination with the mTOR pathways [42]. We report that these agents in combination with castration greatly reduced androgen independence, co-administration being required. In addition, strong support come from a study where everolimus combined with an aromatase inhibitor improved progression-free survival in patients with hormone-receptor–positive advanced breast cancer previously treated with nonsteroidal aromatase inhibitors [43].

Our data confirm that AR remains a central actor in PCa cell survival, and our data suggest that androgen receptor can be activated by alternative pathways resulting in androgen independence. Finally, we showed that combination treatment of castration with targeted molecular therapy significantly reduced the risk of hormone escape. This study might have great implication in the treatment of androgen dependent prostate cancer and dual inhibition is worth exploring in the clinical setting.

## Supporting Information

Figure S1 **Schematic representation of the** ***in vivo*** **treatment regimens.**
(TIF)

Figure S2 **Relative mRNA expression of androgen receptor (AR) (black bars) and intratumoral prostate-specific antigen (PSA) (white bars) in androgen independent (AI) variants.** *indicates variants with amplified *AR* gene copy number. Abbreviations used to identify variants are explained in the legend of Figure 2.
(TIF)

Figure S3 **Hormone response profile in the AIde-a4 castration-resistant tumor.** The responsiveness of the AR in the AIde-a4 castration-resistant tumor was assessed by using the yeast-based functional assay and compared with that of the wild-type AR as described in Material and Methods. Histograms represent aberrant responses obtained with 100 nM β-estradiol (E2) and 1 µM cyproterone acetate (CPA) when compared with the wild-type AR.
(TIF)

Figure S4 **Constitutive activity of Q693X androgen receptor variant.** The nonsense mutation Q693X leads to a truncated androgen receptor variant, which is deleted of the entire ligand-binding domain and AF-2. When compared to the ligand-dependent activity of the wild-type AR, the Q693X AR variant demonstrated constitutive activity in the yeast-based AR assay. Histograms represent the relative number of colonies obtained in the presence of the indicated hormone or anti-androgen to that obtained in the presence of 100 nM DHT. *DHT, dihydrotestosterone; And, androstenedione; Prog, progesterone; Bic, bicalutamide; Flut, flutamide.*
(TIF)

Table S1 **Number of mice assigned to each treatment group.**
(DOC)

Table S2 ***In vivo*** **PAC120 tumor response to different hormone treatments.**
(DOC)

Table S3 **Results of the comparative genomic hybridization array of DNA from androgen-independent (AI) tumors.** Minimal regions were altered in more than 20% of AI variants. *Regions on 1 p, 19 p and 19 q were associated with a delayed time to hormone escape.
(DOC)

Table S4 **Characteristics of the androgen-independent (AI) variants.** Data for AIde01 to AIde04 were obtained from independent experiments (data not shown). Data for mRNA expression (androgen receptor, AR) and tumoral prostate-specific antigen (PSA), circulating PSA levels and Protein expression (see last column) are relative to their mean expression in PAC120 tumors (units are arbitrary).
(DOC)

Methods S1 (DOC)
