# Supplementary figures and images for: Risk of Hormone Escape in a Human Prostate Cancer Model Depends on Therapy Modalities and Can Be Reduced by Tyrosine Kinase Inhibitors
Source: PLoS One. 2012 Aug 6;7(8):e42252. doi: 10.1371/journal.pone.0042252 (PMC3412862; doi:10.1371/journal.pone.0042252)

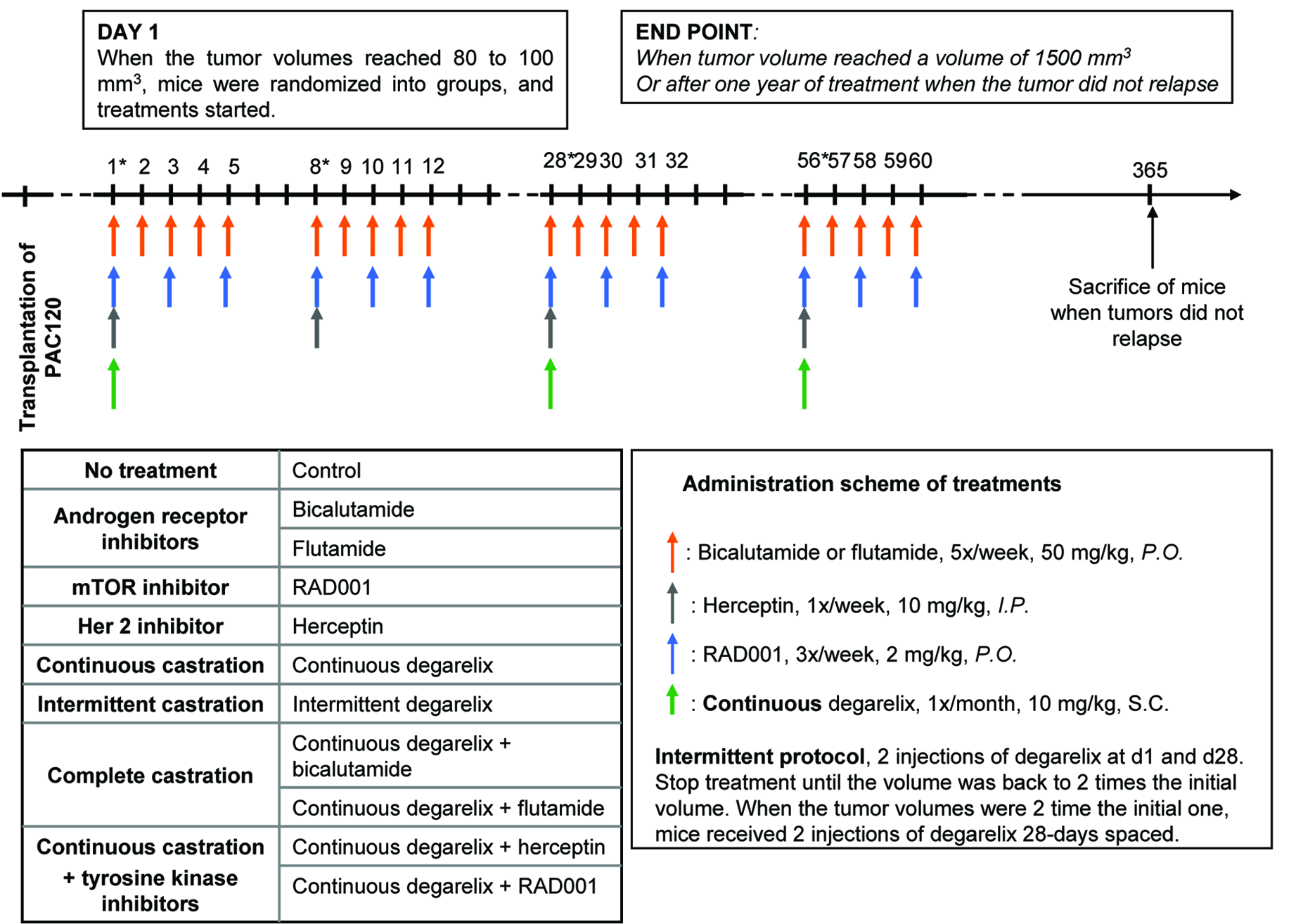

Supplement: Figure S1 — Schematic representation of the in vivo treatment regimens. (TIF) [file pone.0042252.s001.tif]

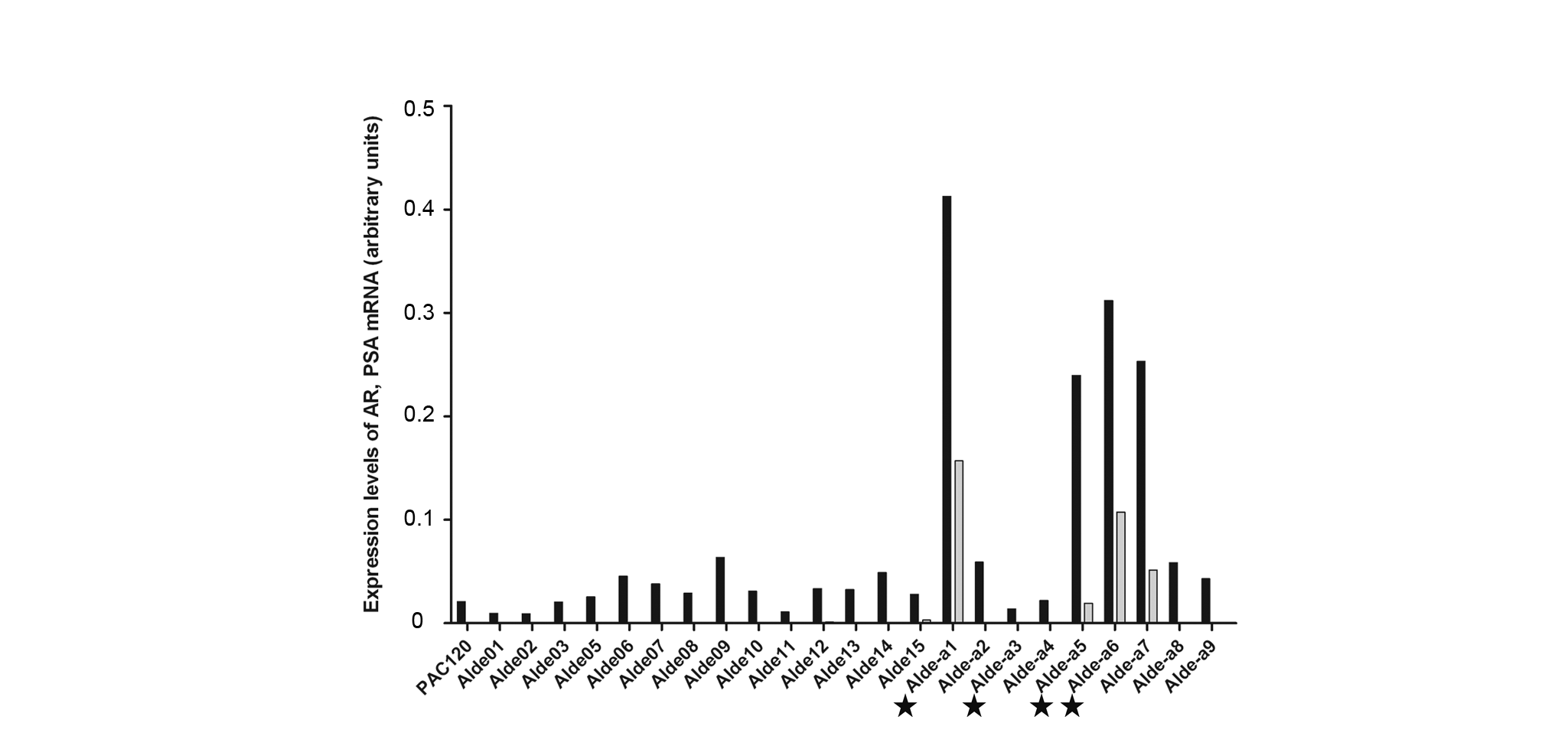

Supplement: Figure S2 — Relative mRNA expression of androgen receptor (AR) (black bars) and intratumoral prostate-specific antigen (PSA) (white bars) in androgen independent (AI) variants. *indicates variants with amplified AR gene copy number. Abbreviations used to identify variants are explained in the legend of Figure 2. (TIF) [file pone.0042252.s002.tif]

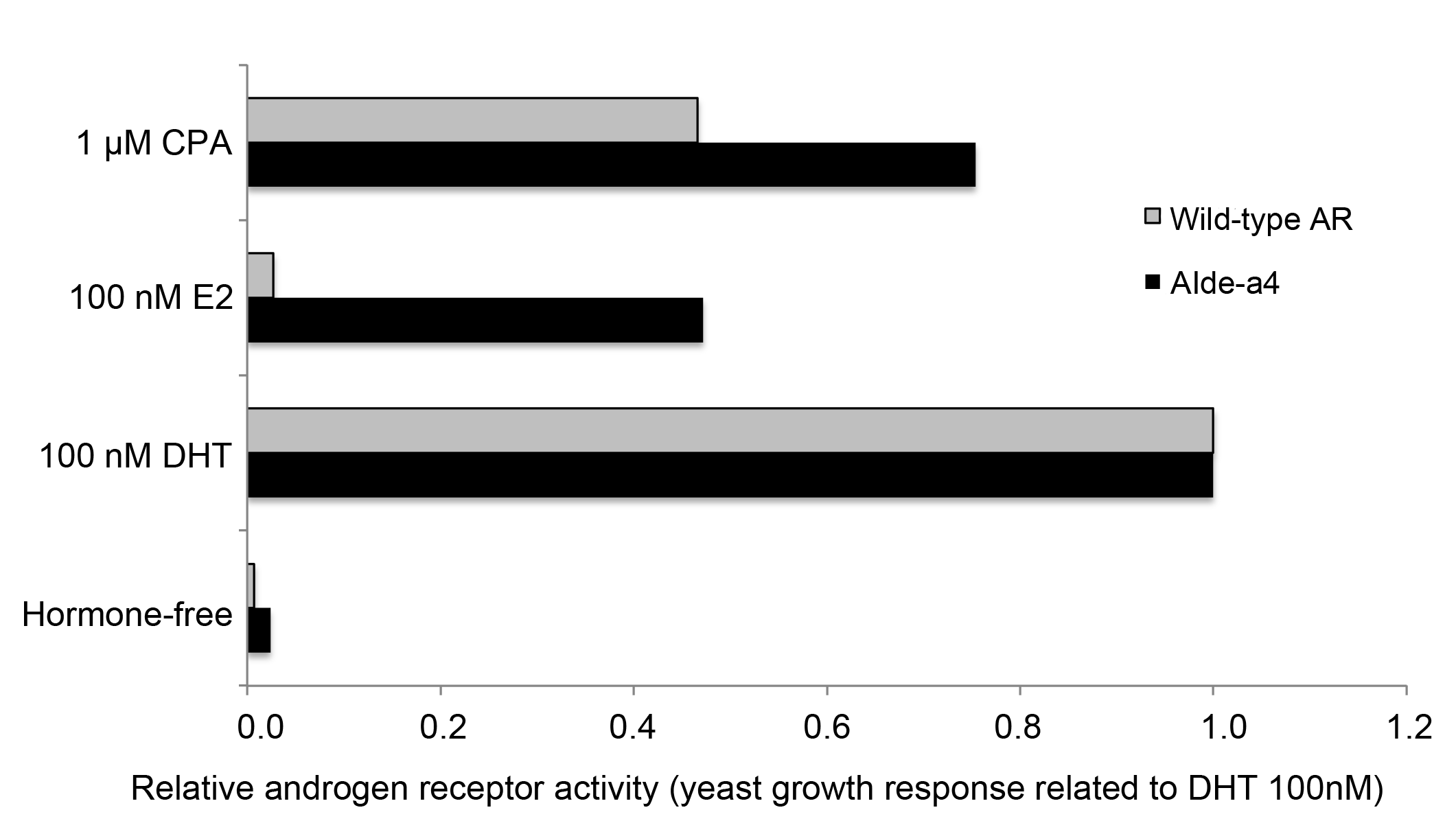

Supplement: Figure S3 — Hormone response profile in the AIde-a4 castration-resistant tumor. The responsiveness of the AR in the AIde-a4 castration-resistant tumor was assessed by using the yeast-based functional assay and compared with that of the wild-type AR as described in Material and Methods. Histograms represent aberrant responses obtained with 100 nM β-estradiol (E2) and 1 µM cyproterone acetate (CPA) when compared with the wild-type AR. (TIF) [file pone.0042252.s003.tif]

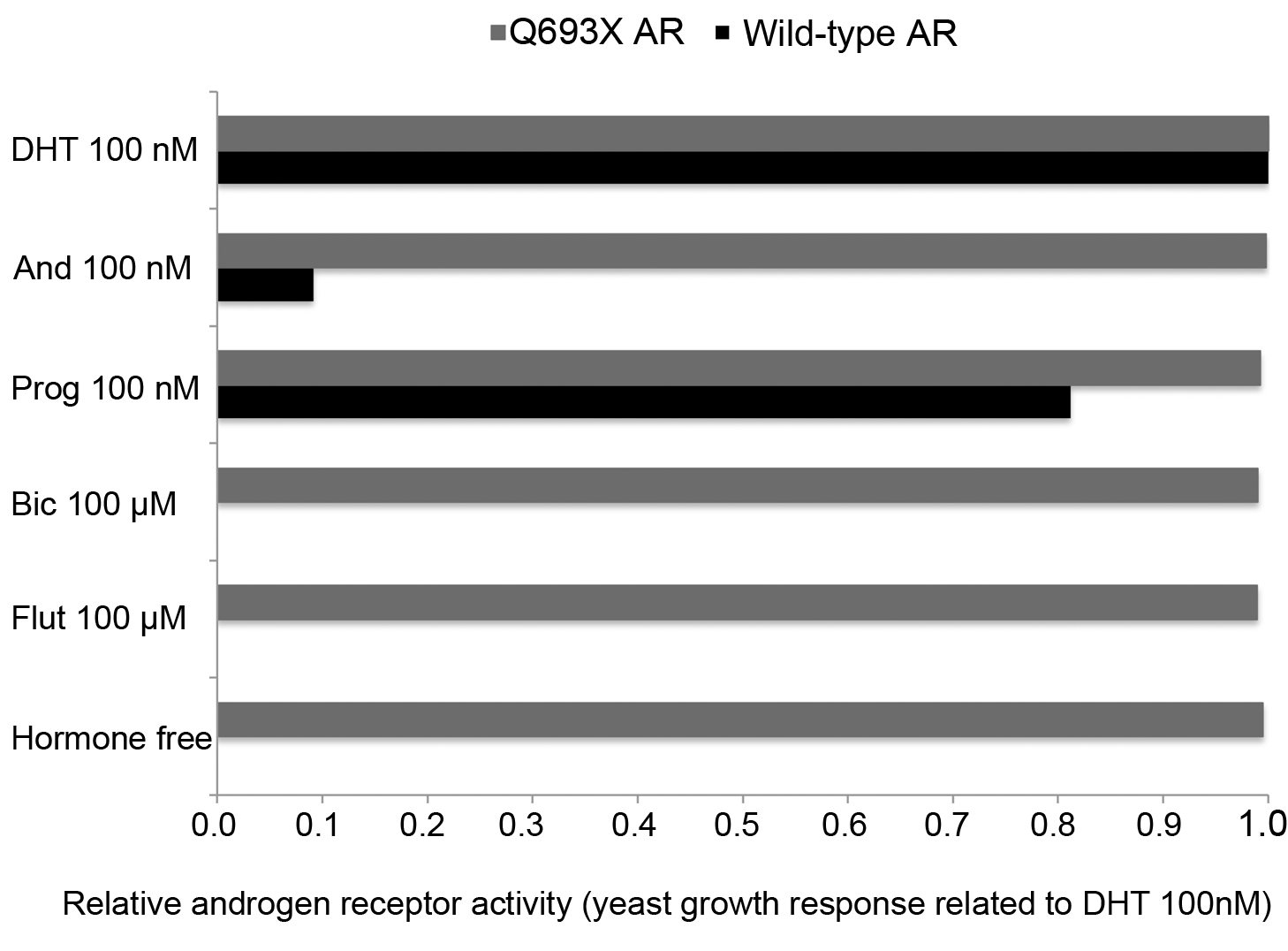

Supplement: Figure S4 — Constitutive activity of Q693X androgen receptor variant. The nonsense mutation Q693X leads to a truncated androgen receptor variant, which is deleted of the entire ligand-binding domain and AF-2. When compared to the ligand-dependent activity of the wild-type AR, the Q693X AR variant demonstrated constitutive activity in the yeast-based AR assay. Histograms represent the relative number of colonies obtained in the presence of the indicated hormone or anti-androgen to that obtained in the presence of 100 nM DHT. DHT, dihydrotestosterone; And, androstenedione; Prog, progesterone; Bic, bicalutamide; Flut, flutamide. (TIF) [file pone.0042252.s004.tif]
