# Supplementary material for: Risk of Hormone Escape in a Human Prostate Cancer Model Depends on Therapy Modalities and Can Be Reduced by Tyrosine Kinase Inhibitors
Source: PLoS One. 2012 Aug 6;7(8):e42252. doi: 10.1371/journal.pone.0042252 (PMC3412862; doi:10.1371/journal.pone.0042252)
Supplement: Table S1 — Number of mice assigned to each treatment group. (DOC) [file pone.0042252.s005.doc]

**Table S1: Number of mice assigned to each treatment group**

|  | **Treatment** | **Number of animals, *n*** |
| --- | --- | --- |
| **1^st^ experiment** | Control | 26 |
|  | Flutamide | 11 |
|  | Bicalutamide | 15 |
|  | Continuous degarelix | 20 |
|  | Intermittent degarelix | 14 |
|  | Flutamide plus degarelix | 10 |
|  | Bicalutamide plus degarelix | 30 |
|  | Trastuzumab plus degarelix | 24 |
| **2^nd^ experiment** | Control | 10 |
|  | Everolimus | 10 |
|  | Continuous degarelix | 18 |
|  | Everolimus plus degarelix | 15 |
