# Supplementary material for: Risk of Hormone Escape in a Human Prostate Cancer Model Depends on Therapy Modalities and Can Be Reduced by Tyrosine Kinase Inhibitors
Source: PLoS One. 2012 Aug 6;7(8):e42252. doi: 10.1371/journal.pone.0042252 (PMC3412862; doi:10.1371/journal.pone.0042252)
Supplement: Table S2 — In vivo PAC120 tumor response to different hormone treatments. (DOC) [file pone.0042252.s006.doc]

**Table S2: *In vivo* PAC120 tumor response to different hormone treatments**

| **Treatment** | **Number of animals with recurrent tumors of total numbers** | **Median time to hormone escape in days [range]** | **Relative risk of tumor recurrence [95% CI]** | ***P* value** |
| --- | --- | --- | --- | --- |
| degarelix (continuous) 1^st^ exp | 8/20 | 274 [211-323] | / | / |
| degarelix (intermittent) | 9/16 | 190 [99-218] | 14.5 [2.98-70.9] | 4x10^-5^ |
| degarelix plus bicalutamide | 24/30 | 197 [71-239] | 6.55 [2.41-17.8] | 3x10^-5^ |
| degarelix plus flutamide | 8/11 | 253 [43-337] | 1.35 [0.48-3.76] | 0.6 |
| degarelix (continuous) 2^nd^ exp | 12/18 | 315 [224-335] | 0.42 [0.15-1.15] | 0.09 |
| degarelix plus trastuzumab | 4/24 | 351 [218-351] | 0.17 [0.04-0.67] | 0.005 |
| degarelix plus everolimus | 0/15 | na | na | <0.001 |

**na: Not applicable**
