# Supplementary material for: Risk of Hormone Escape in a Human Prostate Cancer Model Depends on Therapy Modalities and Can Be Reduced by Tyrosine Kinase Inhibitors
Source: PLoS One. 2012 Aug 6;7(8):e42252. doi: 10.1371/journal.pone.0042252 (PMC3412862; doi:10.1371/journal.pone.0042252)
Supplement: Table S3 — Results of the comparative genomic hybridization array of DNA from androgen-independent (AI) tumors. Minimal regions were altered in more than 20% of AI variants. *Regions on 1 p, 19 p and 19 q were associated with a delayed time to hormone escape. (DOC) [file pone.0042252.s007.doc]

**Table S3: Results of the comparative genomic hybridization array of DNA from androgen-independent (AI) tumors**

|  | **Chromosome** | **Cytogenetic region** | **Position start (bp)** | **Position end (bp)** | **Frequency of alteration (%)** |
| --- | --- | --- | --- | --- | --- |
| *Gains* | 1 | 1p36.3 | 2062390 | 3536847 | 34.6 |
|  | 1 | 1p36.3***** | 5629490 | 6963427 | 26.9 |
|  | 1 | 1p36.2-1p36.1***** | 7414435 | 23210893 | 26.9 |
|  | 2 | 2p25-2p12 | 8101 | 80149809 | 23.1 |
|  | 6 | 6p25 | 143430 | 356931 | 19.2 |
|  | 9 | 9q33-9q34 | 124698077 | 140118658 | 19.2 |
|  | 16 | 16p13.3 | 1549281 | 2410889 | 50.0 |
|  | 16 | 16p12-16p11.2 | 27477041 | 32266552 | 26.9 |
|  | 16 | 16p11.2 | 31482938 | 33811616 | 26.9 |
|  | 19 | 19p13.3-19p13.2***** | 209335 | 11023365 | 23.1 |
|  | 19 | 19p12-19q12 | 24107998 | 33184598 | 23.1 |
|  | 19 | 19q12-19q13.2***** | 35023269 | 46374217 | 23.1 |
|  | 19 | 19q13.2-19q13.3***** | 46654324 | 47900835 | 23.1 |
| *Losses* | 1 | 1q22-1q23 | 154512748 | 155779905 | 19.2 |
|  | 2 | 2q11.2-2q23 | 95863608 | 151865209 | 30.8 |
|  | 2 | 2q24-2q31 | 165852984 | 173742911 | 19.2 |
|  | 2 | 2q34 | 211961272 | 214242624 | 38.5 |
|  | 2 | 2q35-2q36 | 220167149 | 223498801 | 46.2 |
|  | 2 | 2q36-2q37 | 230589338 | 241673431 | 53.8 |
|  | 4 | 4q12-4q35 | 52570687 | 190673464 | 19.2 |
|  | 7 | 7p11.1-7q11.2 | 57980733 | 61793417 | 30.8 |
|  | 7 | 7q11.2 | 61659285 | 62015372 | 34.6 |
|  | 11 | 11p15 | 12376027 | 19314832 | 23.1 |
|  | 11 | 11p14-11p13 | 27565145 | 32289647 | 19.2 |
|  | 11 | 11p13-11p12 | 34885665 | 37746990 | 23.1 |
|  | 11 | 11p12-11p11.2 | 42892423 | 47997899 | 23.1 |
|  | 11 | 11q13-11q25 | 69070125 | 134430723 | 23.1 |
|  | 15 | 15q15 | 38596512 | 41241173 | 34.6 |
|  | 15 | 15q15-15q21 | 41424332 | 43278952 | 34.6 |
|  | 22 | 22q11.1-22q11.2 | 15779661 | 21020159 | 38.5 |
|  | 22 | 22q11.2-22q13 | 21211157 | 49564810 | 38.5 |
|  | X | Xq12-Xq13 | 66716642 | 70074011 | 19.2 |
|  | X | Xq21 | 88105410 | 92542624 | 26.9 |
|  | Y | Yp11.3-Yp11.2 | 2845503 | 6163980 | 61.5 |
|  | Y | Yq11.2 | 23210738 | 26906386 | 61.5 |

Minimal regions were altered in more than 20% of AI variants. * Regions on 1p, 19p and 19q were associated with a delayed time to hormone escape.
