# Supplementary material for: Risk of Hormone Escape in a Human Prostate Cancer Model Depends on Therapy Modalities and Can Be Reduced by Tyrosine Kinase Inhibitors
Source: PLoS One. 2012 Aug 6;7(8):e42252. doi: 10.1371/journal.pone.0042252 (PMC3412862; doi:10.1371/journal.pone.0042252)
Supplement: Table S4 — Characteristics of the androgen-independent (AI) variants. Data for AIde01 to AIde04 were obtained from independent experiments (data not shown). Data for mRNA expression (androgen receptor, AR) and tumoral prostate-specific antigen (PSA), circulating PSA levels and Protein expression (see last column) are relative to their mean expression in PAC120 tumors (units are arbitrary). (DOC) [file pone.0042252.s008.doc]

**Table S4 Characteristics of the androgen-independent (AI) variants**

|  | **delay to** | **EDT** | **Tumor growth** | | | | **Clustering** | **AR** | **mRNA** | | **Circulating** | **Mutations** | **Protein expression** | | | |
| --- | --- | --- | --- | --- | --- | --- | --- | --- | --- | --- | --- | --- | --- | --- | --- | --- |
|  | **(escape (days)** |  | **latency p1** | **EDT p1** | **latency p3** | **EDT p3** | **group** | **amplification** | **AR** | **tumoral PSA** | **PSA** |  | **AR** | **Her2** | **pERK/ERK** | **pAKT/AKT** |
| PAC120 | / | / | / | / | / | / | / | no | 1.0 | 1.0 | 1.0 | nd | 1.0 | 1.0 | 1.0 | 1.0 |
| AIde01 | 208 | - | 78 | 17 | 30 | 7 | 1 | no | 0.5 | 0.1 | nd | nd | 2.6 | 7.4 | 6.0 | 0.8 |
| AIde02 | 208 | - | - | - | 37 | 8 | 3 | no | 0.4 | 0.0 | nd | nd | nd | nd | nd | nd |
| AIde03 | 178 | - | 29 | 28 | 173 | 11 | 3 | no | 1.0 | 0.1 | 0.3 | nd | nd | nd | nd | nd |
| AIde04 | 172 | 21 | 196 | - | 31 | 10 | 1 | no | nd | nd | nd | nd | nd | nd | nd | nd |
| AIde05 | 211 | 14 | 104 | 14 | nd | nd | 4 | no | 1.2 | 0.0 | 1.1 | nd | 1.4 | 9.6 | 3.3 | 0.7 |
| AIde06 | 169 | 18 | 160 | 7 | 61 | 13 | 4 | no | 2.1 | 0.1 | 0.7 | nd | 0.8 | 8.6 | 3.6 | 1.4 |
| AIde07 | 162 | 14 | 126 | 14 | 39 | 10 | 4 | no | 1.8 | 0.0 | 0.3 | nd | 2.0 | 10.4 | 1.5 | 0.4 |
| AIde08 | 225 | 10 | 134 | 23 | nd | nd | 4 | no | 1.4 | 0.1 | nd | mutated | 0.7 | 10.2 | 7.6 | 1.3 |
| AIde09 | 232 | 13 | 190 | 7 | 27 | 7 | 4 | no | 3.0 | 0.1 | nd | nd | 0.5 | 6.5 | 3.6 | 1.1 |
| AIde10 | 232 | 11 | 92 | 33 | nd | nd | 2 | no | 1.5 | 0.1 | 5.3 | wt | nd | nd | nd | nd |
| AIde11 | 281 | 18 | 116 | 13 | 41 | 10 | 2 | no | 0.5 | 0.1 | 0.4 | wt | 0.6 | 8.0 | 2.0 | 2.1 |
| AIde12 | 176 | - | - | - | 51 | 11 | 2 | no | 1.6 | 1.7 | nd | nd | 1.0 | 14.4 | 5.5 | 2.4 |
| AIde13 | 260 | 12 | 221 | 16 | 40 | 10 | 5 | no | 1.5 | 0.0 | 0.8 | nd | 1.0 | 7.9 | 0.5 | 2.0 |
| AIde14 | 330 | 10 | 160 | 5 | nd | nd | 5 | no | 2.3 | 0.1 | 2.0 | nd | nd | nd | nd | nd |
| AIde15 | 295 | 8 | 223 | 15 | 33 | 9 | 3 | no | 1.3 | 4.8 | 1.6 | wt | 0.9 | 11.3 | 0.3 | 3.3 |
| AIde-a1 | 212 | 5 | 32 | 7 | 32 | 7 | 2 | amplified | 20 | 272 | 338 | wt | 5.5 | 6.5 | 2.3 | 2.5 |
| AIde-a2 | 240 | 19 | 120 | 27 | nd | nd | 5 | no | 2.8 | 0.0 | 0.4 | mutated | 1.8 | 8.6 | 5.6 | 1.6 |
| AIde-a3 | 120 | 36 | 40 | 11 | 20 | 13 | 2 | amplified | 0.7 | 0.0 | 25 | mutated | nd | nd | nd | nd |
| AIde-a4 | 240 | 15 | 60 | 33 | 35 | 10 | 4 | no | 1.0 | 0.1 | nd | mutated | 0.9 | 8.6 | 6.6 | 11.2 |
| AIde-a5 | 191 | 11 | 48 | 10 | 46 | 10 | 2 | amplified | 11 | 34 | 1.4 | mutated | nd | nd | nd | nd |
| AIde-a6 | 198 | 14 | 76 | 8 | 30 | 8 | 2 | amplified | 15 | 186 | 79 | wt | 2.1 | 5.6 | 0.4 | 3.7 |
| AIde-a7 | 233 | 10 | 36 | 6 | 34 | 8 | 5 | outlier | 12 | 89 | 58 | mutated | 2.8 | 5.3 | 2.8 | 0.8 |
| AIde-a8 | 226 | 12 | 60 | 17 | 31 | 8 | 1 | no | 2 | 0.3 | 0.3 | wt | nd | nd | nd | nd |
| AIde-a9 | 296 | 14 | 134 | 7 | 20 | 10 | 5 | no | 2.8 | 0.1 | 0.2 | wt | nd | nd | nd | nd |
| AIde-t1 | 211 | 4 | 89 | 14 | 36 | 14 | 1 | no | nd | nd | nd | wt | 1.3 | 3.6 | 2.0 | 2.2 |
| AIde-t2 | 358 | 10 | - | - | 55 | 11 | 5 | no | 1.3 | 0.1 | 1.1 | mutated | 1.6 | 5.8 | 0.3 | 1.2 |
