# Supplementary material for: Risk of Hormone Escape in a Human Prostate Cancer Model Depends on Therapy Modalities and Can Be Reduced by Tyrosine Kinase Inhibitors
Source: PLoS One. 2012 Aug 6;7(8):e42252. doi: 10.1371/journal.pone.0042252 (PMC3412862; doi:10.1371/journal.pone.0042252)
Supplement: Methods S1 — (DOC) [file pone.0042252.s009.doc]

**Supplementary methods**

Transplantation of AI variants

In the group of AI variants obtained after degarelix treatment, the proportion of successful tumor transplants (‘tumor take’) was 63% and thus 15 AI variants were produced after this regimen (denoted AIde). The tumor take in transplanted mice was 72% when tumors recurred after complete androgen blockade; 9 tumors were AI after complete blockade (denoted AIde-a). Regardless of type of hormone treatment, the time to first passage was prolonged as compared with subsequent passages (Supplementary table 1, available online).

Immunohistochemical studies

Light microscope histology studies used 4-µm-thick formalin fixed paraffin-embedded sections from PAC120, PAC120 residual tumors and AI variants stained with hematoxylin and eosin safran.

Total PSA assay

We collected serum when mice were sacrificed to measure total PSA levels using an automated quantitative enzyme-linked fluorescent immunoassay developed on the VIDAS analyzer (bioMerieux, Marcy-l’Etoile, France). The assay was performed according to the manufacturer recommendations and had a sensitivity of 0.019 ng/mL. Each sample was assayed twice.

RT-PCR probes

Using TRIzol reagent (Invitrogen, Cergy Pontoise, France). Relative gene expression was analyzed by two-step RT-PCR using Power SYBR Green PCR Master Mix (Applied Biosystems, Courtaboeuf, France). PCR were run on a 7300 Real-Time PCR System (Applied Biosystems). Fifty cycles of PCR (95°C, 15 sec; 60°C, 1 min) were used for quantification of all genes. Human Cyclophilin A was used as an internal control.

| Cyclophilin A | Forward | GTCAACCCCACCGTGTTCTT |
| --- | --- | --- |
|  | Reverse | CTGCTGTCTTTGGGACCTTGT |
| AR | Forward | GCCATTGAGCCAGGTGTAGTG |
|  | Reverse | TGGAGTTGACATTGGTGAAGGA |
| PSA | Forward | GTGGGTCCCGGTTGTCTTC |
|  | Reverse | CCACAATCCGAGACAGGATGA |

Western-blotting

Tumors were lysed in laemmli buffer.^1^ Proteins were resolved on reducing 4% to 12% SDS polyacrylamide gels and transferred to nitrocellulose membranes (Bio-Rad, Marnes la Coquette, France). Membranes were saturated with Tris-Buffered Saline Tween-20 – Bovine Serum Albumin (TBST-BSA) and incubated overnight at 4°C with primary antibodies. After washes, membranes were incubated with horseradish peroxidase secondary antibodies (Dako, Trappes, France) for 1 h at room temperature. Bound antibodies were visualized with a chemoluminescent detection system (ECL; Amersham Pharmacia Biotech, Orsay, France). Quantification was performed using a LAS-3000 Luminescent Image analyzer and Image Gauge software (Fuji, FSVT, Courbevoie, France).

Array CGH

A genome-wide resource of 5,244 fluorescence in situ hybridization–mapped sequenced bacterial artificial chromosome (BAC) and P1-derived artificial chromosome (PAC) clones were represented as immobilized DNA targets on glass slides, allowing a mean resolution of 0.5 Mb all along the genome. Each clone was spotted in quadruplicate on slide prepared by Integragen^TM^ (Evry, France). 1.5 µg of DNA sample were digested with DpnII enzyme (Ozyme), and labeled by random priming using a Bioprime DNA labeling kit (Invitrogen) with the appropriate cyanine dye (Cy3 or Cy5; Perkin-Elmer). The control and test DNAs were co-precipitated with Cot-1 DNA (Invitrogen), denatured, and resuspended in hybridization buffer. After a 24 h hybridization, slides were washed with SDS and saline citrate, dried and scanned with a GenePix 4000B scanner (Axon Instruments Inc., Union City, CA, USA)*.* Image analysis was done with Genepix 5.1 software (Axon).^2^ Any BAC with less than two replicates flagged for not fulfilling qualitative spot criteria was excluded. The MANOR algorithm was used to perform normalization.^3^ Spots showing a low signal-to-noise ratio or poor replicate consistency were discarded.

Status assignment (loss, normal, gain and amplification of chromosome copy number) was performed by using the GLAD algorithm.^4^ Minimal regions of amplification supported by at least one tumor were detected. Subsequently, minimal regions of amplification, gain or loss supported by at least 20% of the total number of tumors were analyzed. The visualization of the data, the computation of the minimal regions and clustering were done using the VAMP software.^2^

Statistical analysis

The Mann-Whitney test was used to assess differences on RTV between two treatments. Kaplan-Meier survival plots and log-rank tests were used to describe and test the differences in time to escape between the different treatment groups. Relative risks (RR) and their 95% confidence intervals (CI) were derived from a Cox proportional-hazards regression model. For Kaplan Meier analysis, the *p* values and median time were calculated until day 351. The Fisher’s exact test was used to assess the difference in frequency of alterations of a given minimal region between two treatments. In case of multiple tests, Benjamini and Hochberg correction was applied.^5^ *P* values under 5% were considered significant for all analyses.

**REFERENCES**

1. Marty B, Maire V, Gravier E, et al: Frequent PTEN genomic alterations and activated phosphatidylinositol 3-kinase pathway in basal-like breast cancer cells. Breast Cancer Res 10:R101, 2008

2. La Rosa P, Viara E, Hupe P, et al: VAMP: visualization and analysis of array-CGH, transcriptome and other molecular profiles. Bioinformatics 22:2066-73, 2006

3. Neuvial P, Hupe P, Brito I, et al: Spatial normalization of array-CGH data. BMC Bioinformatics 7:264, 2006

4. Hupe P, Stransky N, Thiery JP, et al: Analysis of array CGH data: from signal ratio to gain and loss of DNA regions. Bioinformatics 20:3413-22, 2004

5. Benjamini Y, Drai D, Elmer G, et al: Controlling the false discovery rate in behavior genetics research. Behav Brain Res 125:279-84, 2001
